# Risk factors for thrombosis in tuberculosis patients admitted to a tuberculosis-dedicated intensive care unit: a retrospective cohort study

**DOI:** 10.3389/fmed.2026.1877596

**Published:** 2026-06-30

**Authors:** Aifeng Liu, Yuewen Qiu, Hongmei Chen, Xiaohua Ma

**Affiliations:** 1 Department of Pathology, The Affiliated Changsha Central Hospital, Hengyang Medical School, University of South China, Changsha, Hunan, China; 2 Department of Ultrasound Medicine, The Affiliated Changsha Central Hospital, Hengyang Medical School, University of South China, Changsha, Hunan, China; 3 Department of Laboratory Medicine, Xinjiang Second Medical College, Karamay, Xinjiang, China; 4 Department of Laboratory, The Affiliated Changsha Central Hospital, Hengyang Medical School, University of South China, Changsha, Hunan, China

**Keywords:** age-stratified analysis, intensive care unit, risk factors, thrombosis, tuberculosis

## Abstract

**Background:**

Patients with tuberculosis in the intensive care unit (ICU) face an elevated risk of thrombosis; however, the contributing factors remain incompletely understood. This study aimed to identify independent risk factors for thrombus formation, evaluate the incremental predictive value of inflammatory biomarkers [C-reactive protein (CRP), D-dimer (DDR), and interleukin-6 (IL-6)], and explore the determinants of IL-6 levels in critically ill tuberculosis patients.

**Methods:**

This retrospective cohort study consecutively enrolled 168 tuberculosis patients admitted to the ICU, including 101 with thrombosis and 67 without. Demographic, clinical, and laboratory data were collected. Univariate and multivariable binary logistic regression analyses were performed to identify independent risk factors. The area under the receiver operating characteristic curve (AUC) and DeLong test were used to compare five logistic models: a base model [age, activated partial thromboplastin time(APTT), and non-TB bacterial/fungal infection status] and four extended models incorporating ln-transformed CRP, DDR, or IL-6, individually or in combination. Multivariable linear regression analysis was employed to identify factors independently associated with IL-6 levels.

**Results:**

Advanced age (OR = 1.057, *p* = 0.001) and prolonged APTT (OR = 1.053, *p* = 0.041) were independent predictors of thrombus formation. In the multivariable model, fungal co-infection (OR = 3.185, *p* = 0.006) and non-TB bacterial co-infection (OR = 0.336, *p* = 0.038) also reached statistical significance. Age-stratified analysis revealed that among patients aged <70 years, only age was independently associated with thrombosis (OR = 1.050, *p* < 0.05), whereas no other parameter demonstrated independent predictive value. The base model yielded an AUC of 0.753; the addition of CRP, DDR, or IL-6 did not produce a statistically significant improvement in discriminatory performance (all *p* > 0.05). In multivariable linear regression, only CRP was independently associated with IL-6 levels (*β* = 0.423, *p* < 0.001).

**Conclusion:**

Age and APTT are independent risk factors for thrombus formation in patients with tuberculosis. Fungal co-infection confers additional thrombotic risk, whereas non-TB bacterial co-infection exhibits a paradoxical protective effect, the underlying mechanisms of which warrant further investigation. A parsimonious model based on age, APTT, and co-infection status demonstrated moderate predictive accuracy (AUC = 0.753), and the incorporation of CRP, DDR, or IL-6 failed to enhance its discriminatory ability. CRP serves as a reliable surrogate marker for IL-6-driven inflammation in this population. Large-scale prospective studies are warranted to validate and extend these findings.

## 1 Introduction

Critically ill patients with tuberculosis remain a major contributor to fatal disease burden worldwide, a problem that is particularly acute in regions with limited healthcare resources (1, 2). These patients commonly exhibit a hypercoagulable profile, rendering venous thromboembolism (VTE) an increasingly prominent clinical concern (3–5). Current data indicate that the incidence of thrombosis during hospitalization among tuberculosis patients is approximately 4% (6, 7), a proportion that may rise substantially in those admitted to the intensive care unit (ICU). Given that thrombotic events not only prolong hospital stays but also elevate mortality risk, early identification and prophylactic strategies have become clinical priorities.

The pathophysiological mechanisms underlying tuberculosis-associated thrombosis are multifaceted, encompassing systemic inflammatory storms, endothelial dysfunction, coagulation cascade disturbances, and prolonged immobilization (8, 9). Although previous studies have elucidated VTE risk factors in the general tuberculosis population, specific data pertaining to the critically ill tuberculosis subgroup in the ICU remain scarce. While advanced age, prolonged APTT, and elevated DDR levels are routinely assessed in clinical practice, their independent contributions to thrombotic risk have yet to be fully elucidated. Furthermore, whether inflammatory biomarkers such as CRP, DDR, and IL-6 can provide incremental predictive value for thrombosis in this specific population has not been systematically evaluated.

As a central mediator of the acute inflammatory response, IL-6 plays a pivotal role in procoagulant and proinflammatory processes (10, 11). Elucidating the independent determinants of IL-6 levels may therefore facilitate the identification of high-risk individuals susceptible to inflammation-driven complications. However, in the context of tuberculosis infection, it remains unclear whether routine markers such as CRP are sufficient to serve as surrogates for IL-6.

Accordingly, this retrospective cohort study aimed to: (1) identify independent risk factors for thrombus formation among tuberculosis patients admitted to the ICU; (2) investigate whether the integration of CRP, DDR, or IL-6 into a baseline prediction model could enhance predictive performance; and (3) determine the independent correlates of IL-6 levels, with particular emphasis on the potential role of CRP.

## 2 Materials and methods

### 2.1 Study design and population

**Figure 1:**
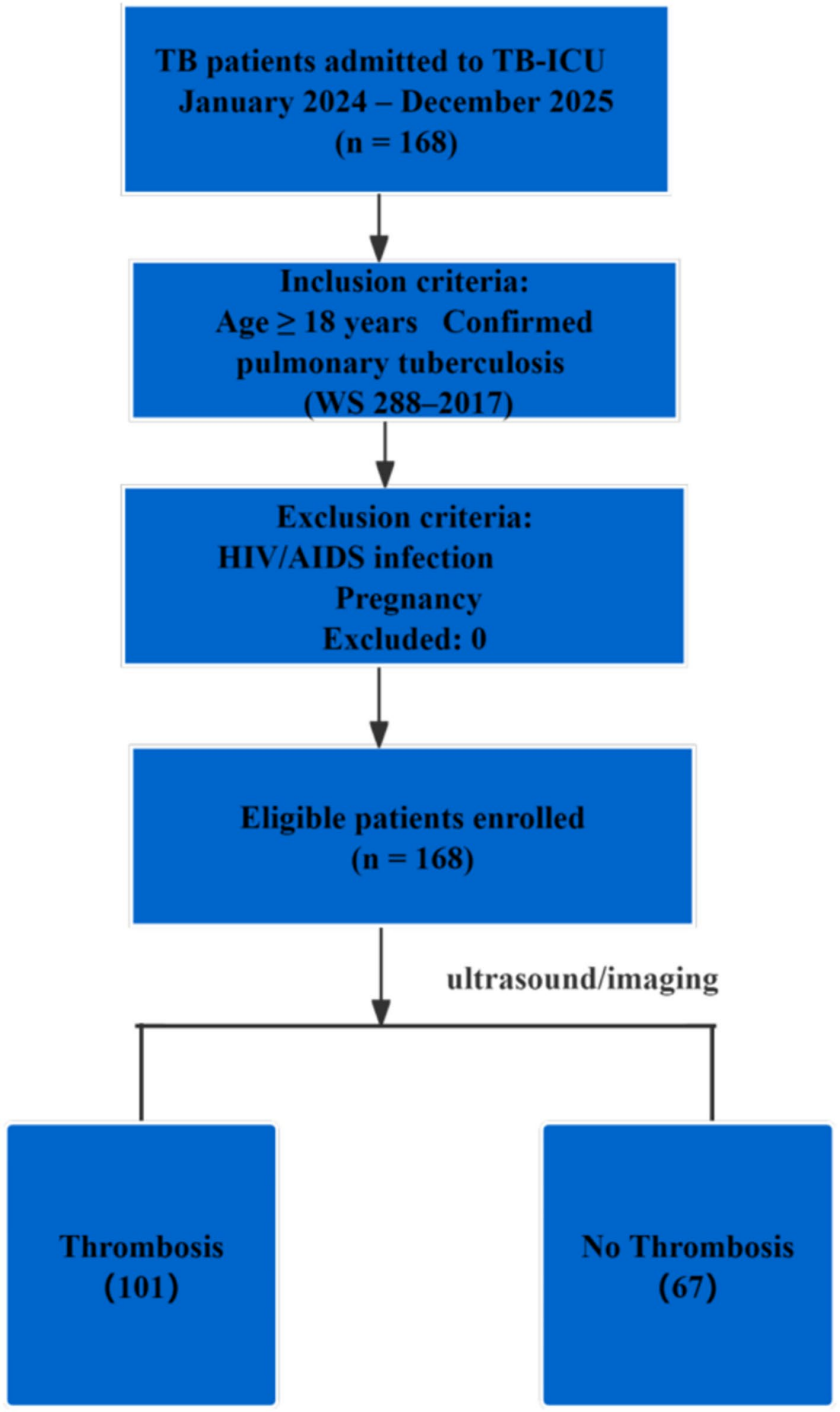
Study flowchart.

A total of 168 consecutive patients with tuberculosis admitted to the Tuberculosis Intensive Care Unit (TB ICU) of our hospital between January 2024 and December 2025 were enrolled in this study. According to the “WS 288–2017” diagnostic standard for tuberculosis (12), each case of pulmonary tuberculosis was confirmed based on a combination of clinical signs, imaging features, and at least one positive laboratory test (i.e., culture, acid-fast bacillus smear, or nucleic acid amplification test for *M. tuberculosis*). Patients were eligible for inclusion if they were aged 18 years or older and had confirmed pulmonary tuberculosis. Patients with HIV/AIDS infection and pregnant women were excluded. Patients were divided into two groups based on the presence or absence of thrombosis confirmed by ultrasound/imaging (Figure 1). This study was reviewed and approved by the Ethics Committee of Changsha Central Hospital (Approval No: 2026 Medical Review No. 108). Due to its retrospective nature, the requirement for informed consent was waived.

### 2.2 Data collection

Demographic and clinical characteristics were extracted from electronic medical records, including age, gender, comorbidities [pulmonary cavitation, extrapulmonary tuberculosis, non-TB bacterial/fungal/viral infection, tumor, chronic obstructive pulmonary disease (COPD), coronary heart disease, hypertension, and diabetes mellitus], and laboratory parameters. Due to the diversity of individual pathogens and the limited number of cases per species, coinfections were analyzed by category (fungal, non-tuberculous bacterial, and viral) rather than at the species level.

Thrombotic events were defined as deep vein thrombosis (DVT) of the lower extremities diagnosed by the attending physician and confirmed by vascular ultrasound using a GE LOGIQ S8 ultrasound system. Pulmonary embolism, arterial thrombosis, and catheter-related thrombosis were not included in this analysis. Thrombus detection was performed during hospitalization, triggered by clinical symptoms suggestive of thrombosis (e.g., leg swelling, chest pain, dyspnea) or abnormally elevated D-dimer levels on laboratory testing.

### 2.3 Laboratory tests

Venous blood samples were collected within 24 h of ICU admission. Complete blood count (neutrophils, platelets, monocytes, lymphocytes) was measured using an automated hematology analyzer (Sysmex XN-9000, Sysmex Corporation). Coagulation parameters, including APTT, prothrombin time (PT), thrombin time (TT), and fibrinogen (FIB), were determined using a standard coagulation analyzer (CS-5100, Sysmex Corporation). DDR and CRP were measured by immunoturbidimetry (Meikang Biotechnology), and IL-6 was detected by chemiluminescence immunoassay (Beijing Hotgen Biotech Corporation). All tests were performed in strict accordance with the manufacturers’ instructions.

### 2.4 Statistical analysis

Statistical analyses were performed using SPSS version 18.0 (SPSS Inc., Chicago, IL, USA) and MedCalc version 23.5 (MedCalc Software Ltd., Ostend, Belgium). A two-tailed *p* value < 0.05 was considered statistically significant. Continuous variables were expressed as median (interquartile range, IQR) and compared using the Mann–Whitney U test. Categorical variables were analyzed using the chi-square test. Normality of continuous data was assessed by the Shapiro–Wilk test. Univariate and multivariate binary logistic regression analyses were used to identify risk factors for thrombosis. Variables with *p* < 0.10 in the univariate analysis were included in the multivariate model to avoid omitting potentially important confounders. The AUC and DeLong test were used to compare the performance of five logistic models. For the determinants of IL-6 levels, variables selected by Spearman correlation analysis (*p* < 0.10) were entered into a multivariate linear regression model with ln_IL-6 as the dependent variable.

## 3 Results

### 3.1 Baseline characteristics of the study population

A total of 168 patients with tuberculosis were enrolled, including 67 patients without evidence of thrombosis (Group A) and 101 patients with documented thrombosis (Group B). The demographic and clinical characteristics of the two groups are summarized in Table 1. Compared with the non-thrombosis group, the median age in the thrombosis group was significantly higher (72 [IQR 63–78] vs. 61 [IQR 52–70] years, Z = −4.575, *p* < 0.001). There were no significant differences between the two groups in terms of gender, pulmonary cavitation, extrapulmonary tuberculosis, bacterial infection, fungal infection, viral infection, malignant tumor, COPD, coronary heart disease, hypertension, or diabetes mellitus (all *p* > 0.05).

**Table 1:** Baseline demographic and clinical characteristics of tuberculosis patients with and without thrombosis.

Characteristic	Group A (No thrombosis, *n* = 67)	Group B (Thrombosis, *n* = 101)	Test statistic (Z/χ^2^)	*p* value
Male sex, *n* (%)	52 (77.6)	81 (80.2)	0.163	0.686
Age (years), median [IQR]	61 [52, 70]	72 [63, 78]	−4.575	<0.001***
Pulmonary cavity, *n* (%)	14 (20.9)	20 (19.8)	0.03	0.863
Extrapulmonary tuberculosis, *n* (%)	25 (37.3)	51 (50.5)	2.825	0.093
Bacterial infection, *n* (%)	9 (13.4)	23 (22.8)	2.279	0.131
Fungal infection, *n* (%)	33 (49.3)	40 (39.6)	1.526	0.217
Viral infection, *n* (%)	7 (10.4)	8 (7.9)	0.316	0.574
Tumor, *n* (%)	6 (9.0)	13 (12.9)	0.616	0.433
COPD, *n* (%)	10 (14.9)	16 (15.8)	0.026	0.872
Coronary artery disease, *n* (%)	17 (25.4)	31 (30.7)	0.559	0.455
Hypertension, *n* (%)	29 (43.3)	53 (52.5)	1.362	0.243
Diabetes mellitus, *n* (%)	22 (32.8)	31 (30.7)	0.086	0.770

***p < 0.001. *p* values were calculated using the Mann–Whitney U test for age. Data are presented as median [interquartile range] for age and *n* (%) for categorical variables. Group A: no thrombosis; Group B: thrombosis. *p* values were calculated using Mann–Whitney U test for age and chi-square test for categorical variables. IQR, interquartile range; COPD, chronic obstructive pulmonary disease.

### 3.2 Analysis of laboratory parameters in patients with tuberculosis

The results of laboratory parameters comparison between the two groups are summarized in Table 2. Compared with Group A, the Group B had significantly prolonged APTT (median 36.2 s vs. 32.0 s, Z = −3.335, *p* = 0.001) and PT (13.2 s vs. 12.7 s, Z = −2.129, *p* = 0.033), as well as a higher DDR level (3.60 μg/L vs. 2.10 μg/L, Z = −2.760, *p* = 0.006). The lymphocyte count (LYM) in the thrombosis group showed a decreasing trend (0.53 vs. 0.56 × 10^9^/L, Z = −1.724, *p* = 0.085), but this difference did not reach statistical significance. There were no significant differences between the two groups in the levels of neutrophils (NEUT), platelets (PLT), monocytes (MONO), fibrinogen (FIB), thrombin time (TT), CRP, or IL-6 (all *p* > 0.05).

**Table 2:** Laboratory parameters of tuberculosis patients with and without thrombosis.

Parameter	Group A (No thrombosis, *n* = 67)	Group B (Thrombosis, *n* = 101)	Z	*p* value
NEUT (×10^9^/L)	7.55 [5.30, 10.11]	7.80 [5.16, 11.27]	−0.800	0.424
PLT (×10^9^/L)	194 [135, 276]	190 [112.5, 266.0]	−0.774	0.439
MONO (×10^9^/L)	0.47 [0.26, 0.68]	0.39 [0.17, 0.60]	−1.184	0.236
LYM (×10^9^/L)	0.56 [0.41, 0.94]	0.53 [0.28, 0.84]	−1.724	0.085
FIB (g/L)	3.69 [2.60, 4.81]	3.59 [2.60, 4.48]	−1.200	0.230
APTT (s)	32.0 [27.3, 35.8]	36.2 [29.55, 44.45]	−3.335	0.001**
PT (s)	12.7 [11.70, 14.30]	13.2 [12.3, 14.75]	−2.129	0.033*
TT (s)	16.80 [15.80, 18.30]	17.0 [15.95, 18.80]	−0.640	0.522
CRP (mg/L)	69.80 [32.55, 141.20]	80.60 [42.35, 116.65]	−0.279	0.781
IL-6 (pg/mL)	37.66 [15.23, 97.05]	42.78 [18.35, 95.43]	−0.556	0.579
DDR (μg/L)	2.1 [1.00, 4.40]	3.60 [1.60, 6.75]	−2.760	0.006**

*p < 0.05; **p < 0.01. *p* values were calculated using the Mann–Whitney U test. Data are presented as median [interquartile range]. Group A: no thrombosis; Group B: thrombosis. *p* values were calculated using the Mann–Whitney U test. *p* < 0.05 indicate statistical significance. NEUT, neutrophil count; PLT, platelet count; MONO, monocyte count; LYM, lymphocyte count; FIB, fibrinogen; APTT, activated partial thromboplastin time; PT, prothrombin time; TT, thrombin time; CRP, C-reactive protein; IL-6, interleukin-6.

### 3.3 Risk factors for thrombosis: univariate and multivariate logistic regression analyses

To identify independent risk factors for thrombosis in patients with tuberculosis in ICU, univariate and multivariate binary logistic regression analyses were performed. Variables with *p* < 0.10 in the univariate analysis (age, APTT, lymphocyte count, DDR, extrapulmonary tuberculosis) as well as clinically relevant comorbidities were included in the multivariate model. The results are summarized in Table 3.

**Table 3:** Univariate and multivariate logistic regression analyses of risk factors for thrombosis in patients with tuberculosis.

Variable	Coding	Univariate analysis		Multivariate analysis	
		OR (95% CI)	*p* value	OR (95% CI)	*p* value
Continuous variables					
Age (years)	Per 1-year increase	1.053 (1.026–1.080)	<0.001***	1.057 (1.024–1.091)	0.001**
APTT (s)	Per 1-s increase	1.069 (1.028–1.112)	0.001**	1.053 (1.001–1.106)	0.041*
PT (s)	Per 1-s increase	1.111 (0.966–1.277)	0.140	0.997 (0.911–1.091)	0.947
Lymphocyte count (×10^9^/L)	Per 1-unit increase	0.566 (0.303–1.056)	0.074	0.614 (0.282–1.340)	0.221
DDR (μg/L)	Per 1-μg/L increase	1.079 (1.004–1.160)	0.038*	1.050 (0.970–1.136)	0.227
Categorical variables					
Fungal infection	0 = no, 1 = yes	1.480 (0.794–2.760)	0.217	3.185 (1.399–7.248)	0.006**
Bacterial infection (non-tuberculous)	0 = no, 1 = yes	0.526 (0.227–1.222)	0.135	0.336 (0.120–0.941)	0.038*
Viral infection	0 = no, 1 = yes	1.356 (0.468–3.934)	0.575	1.443 (0.412–5.046)	0.566
Pulmonary cavity	0 = no, 1 = yes	1.070 (0.497–2.301)	0.863	1.129 (0.470–2.712)	0.787
Extrapulmonary tuberculosis	0 = no, 1 = yes	0.584 (0.311–1.096)	0.094	0.620 (0.278–1.383)	0.243
Tumor	0 = no, 1 = yes	0.666 (0.240–1.848)	0.435	0.487 (0.150–1.576)	0.230
COPD	0 = no, 1 = yes	0.932 (0.395–2.199)	0.872	1.314 (0.439–3.937)	0.626
Coronary artery disease	0 = no, 1 = yes	0.768 (0.384–1.537)	0.455	1.385 (0.564–3.400)	0.477
Hypertension	0 = no, 1 = yes	0.691 (0.371–1.287)	0.244	0.794 (0.369–1.709)	0.556
Diabetes mellitus	0 = no, 1 = yes	1.104 (0.569–2.141)	0.770	0.771 (0.332–1.792)	0.545

CI, confidence interval; OR, odds ratio; APTT, activated partial thromboplastin time; PT, prothrombin time; DDR, D-dimer; COPD, chronic obstructive pulmonary disease. *p < 0.05; **p < 0.01; ***p < 0.001. Variables with *p* < 0.10 in the univariate analysis were included in the multivariate model.

In the univariate analysis, older age (OR = 1.053, 95% CI: 1.026–1.080, *p* < 0.001), prolonged APTT (OR = 1.069, 95% CI: 1.028–1.112, *p* = 0.001), and elevated DDR (OR = 1.079, 95% CI: 1.004–1.160, *p* = 0.038) were significantly associated with an increased risk of thrombosis. LYM showed a marginal protective effect (OR = 0.566, *p* = 0.074). Other variables did not reach statistical significance in the univariate analysis.

In the multivariate model, after adjusting for potential confounders, age (OR = 1.057, 95% CI: 1.024–1.091, *p* = 0.001) and APTT (OR = 1.053, 95% CI: 1.001–1.106, *p* = 0.041) remained independent risk factors for thrombosis. Notably, fungal infection and bacterial infection emerged as significant predictors in the multivariate model; bacterial infection exhibited an independent protective effect (OR = 0.336, 95% CI: 0.120–0.941, *p* = 0.038), and its specific mechanism warrants further investigation. In contrast, DDR lost its significance after adjustment (OR = 1.050, *p* = 0.227). Other variables, including PT, LYM, extrapulmonary tuberculosis, and all other comorbidities, had no independent association with thrombosis (all *p* > 0.05).

### 3.4 Impact of age stratification on risk factors for thrombosis in patients

Multivariate logistic regression analysis demonstrated that age and APTT were independent risk factors for thrombosis in tuberculosis patients. Given that age is an important confounding factor and there was a significant difference in age distribution between the two groups, to further clarify whether the association between each indicator and thrombosis is influenced by age, this study further conducted age-stratified analysis with 70 years as the cutoff. The age cut-off of 70 years was chosen *a priori* based on its clinical relevance as a widely accepted threshold for defining older patients and its established role in the Padua Prediction Score for venous thromboembolism risk assessment (13), rather than relying on a data-driven cut-off such as the sample median. The results presented in Table 4 indicated that among patients aged < 70 years, only age was independently associated with thrombosis (OR = 1.050, *p* < 0.05), while no other indicators exhibited independent predictive value. In contrast, among elderly patients aged ≥ 70 years, none of the included indicators had an independent statistical association with thrombosis.

**Table 4:** Analysis of risk factors for thrombosis in tuberculosis patients across different age strata.

Age stratification (Years)	Indicators	B	Odds ratio (OR)	95% Confidence interval (95% CI)	*p* value
< 70	Age	0.049	1.050	1.004–1.097	0.031*
	APTT	0.054	1.055	0.984–1.131	0.131
	PT	0.057	1.058	0.865–1.295	0.582
	CRP	0.000	1.000	0.993–1.007	0.973
	IL-6	−0.004	0.996	0.990–1.002	0.179
	DDR	0.045	1.046	0.951–1.152	0.355
≥ 70	Age	−0.037	0.963	0.893–1.010	0.336
	APTT	0.055	1.057	0.984–1.135	0.128
	PT	0.003	1.003	0.899–1.120	0.954
	CRP	0.000	1.000	0.989–1.012	0.974
	IL-6	0.001	1.001	0.993–1.009	0.838
	DDR	0.062	1.064	0.916–1.235	0.419

B, regression coefficient; OR, odds ratio; CI, confidence interval; APTT, activated partial thromboplastin time; PT, prothrombin time; CRP, C-reactive protein; IL-6, interleukin-6; DDR, D-dimer.*p < 0.05.

### 3.5 Comparison of predictive performance of logistic regression models for thrombosis

To evaluate whether the addition of inflammatory markers (CRP, DDR, IL-6) to the baseline model (age, APTT, bacterial infection, fungal infection) could improve the discriminative ability for thrombosis, five logistic regression models were constructed, and their area under the receiver operating characteristic curve (AUC) were compared. As shown in Table 5, the baseline model (Model 1) had an AUC of 0.753 (95% CI: 0.681–0.816, *p* < 0.001), indicating moderate discriminative ability. After adding ln_CRP alone (Model 2), ln_DDR alone (Model 3), or ln_IL-6 alone (Model 4), the AUC slightly increased to 0.758, 0.758, and 0.756, respectively. The full model (Model 5), which incorporated all three inflammatory markers simultaneously, had an AUC of 0.764 (95% CI: 0.693–0.826). However, the DeLong test demonstrated that there were no statistically significant improvements in the AUC of any extended model compared with the baseline model (all *p* > 0.05; Model 2 vs. baseline model: *p* = 0.070; Model 3: *p* = 0.581; Model 4: *p* = 0.447; Model 5: *p* = 0.293). These findings suggest that in patients with tuberculosis, the addition of CRP, DDR, or IL-6 to the model containing age, APTT, and comorbid infections does not significantly enhance the predictive ability for thrombosis.

**Table 5:** Comparison of predictive performance of logistic regression models for thrombosis.

Model	Variables included	AUC	SE	95% CI	*p* value ^a^	*p* value for comparison with baseline model ^b^
Baseline model 1	Age + APTT + Bacteria + Fungi	0.753	0.0386	0.681–0.816	<0.001***	–
Baseline model 2	Baseline model 1 + ln_CRP	0.758	0.0383	0.686–0.821	<0.001***	0.070
Baseline model 3	Baseline model 1 + ln_DDR	0.758	0.0377	0.686–0.821	<0.001***	0.581
Baseline model 4	Baseline model 1 + ln_IL-6	0.756	0.0384	0.684–0.819	<0.001***	0.447
Baseline model 5	Baseline model 1 + ln_CRP + ln_DDR + ln_IL-6	0.764	0.0372	0.693–0.826	<0.001***	0.293

^a^*p* value for testing whether the AUC is significantly greater than 0.5 (the null hypothesis of no discriminative ability); ^b^*p* value from DeLong’s test for the equality of AUCs between each extended model and the baseline model (null hypothesis: no difference in discriminative ability). ***p < 0.001.

### 3.6 Multivariate linear regression analysis of factors influencing IL-6 levels

To identify independent factors associated with IL-6 levels in patients with tuberculosis, multivariate linear regression analysis was performed with ln_IL-6 as the dependent variable. The independent variables included ln_age, ln_APTT, ln_PT, ln_LYM, ln_DDR, and ln_CRP. As shown in Table 6, among all predictors, only ln_CRP was significantly and independently associated with ln_IL-6 (B = 0.581, 95% CI: 0.385–0.776, standardized coefficient *β* = 0.423, t = 5.869, *p* < 0.001). Other variables, such as ln_age, ln_APTT, ln_PT, ln_LYM, or ln_DDR, did not reach statistical significance (all *p* > 0.05).

**Table 6:** Multivariate linear regression analysis of factors influencing IL-6 levels.

Variable	B (95% CI)	Standard error	Standardized coefficient *β*	t	*p* value
ln_Age	0.007 (−0.005, 0.019)	0.006	0.079	1.129	0.260
ln_APTT	0.012 (−0.007, 0.031)	0.010	0.093	1.221	0.224
ln_PT	0.023 (−0.002, 0.048)	0.013	0.132	1.849	0.066
ln_LYM	−0.022 (−0.360, 0.315)	0.171	−0.009	−0.130	0.897
ln_DDR	0.096 (−0.059, 0.251)	0.078	0.087	1.227	0.221
ln_CRP	0.581 (0.385, 0.776)	0.099	0.423	5.869	<0.001***

Dependent variable: ln_IL-6 (natural logarithm of interleukin-6). Independent variables (ln_Age, ln_APTT, ln_PT, ln_LYM, ln_DDR, ln_CRP) were also natural log-transformed due to skewed distributions. ***p < 0.001.

## 4 Discussion

The risk of thrombosis in tuberculosis has been primarily investigated in general ward settings or mixed cohorts. These studies, including a global systematic review and meta-analysis (6) and a dedicated case–control study (9), have focused on documenting the prevalence and identifying traditional risk factors for venous thromboembolism in hospitalized, but generally non-critically ill, TB patients. In contrast, studies specifically focusing on TB patients admitted to the ICU remain sparse. In the ICU context, our findings confirm the established role of advanced age and identify prolonged APTT as an additional predictor in the overall cohort. However, age-stratified analysis revealed that APTT lost its independent predictive value within each age subgroup, suggesting that its effect may be partially confounded by age. This nuanced relationship between APTT and thrombosis has rarely been explored in non-ICU TB cohorts. Furthermore, the contrasting roles of fungal and bacterial coinfections observed in our study may reflect the higher infectious burden and complexity of TB patients in the ICU, a phenomenon that has rarely been explored in general TB populations. This comparison underscores that thrombotic risk in TB patients admitted to the ICU is driven by a distinct set of factors beyond those identified in less severe cases, reinforcing the need for ICU-specific risk stratification approaches.

The hypercoagulable state in tuberculosis is driven by several interconnected mechanisms, including systemic inflammation-induced activation of the coagulation cascade, upregulation of tissue factor expression by activated monocytes and macrophages, and endothelial activation that promotes platelet adhesion and aggregation. These processes, combined with prolonged immobilization and impaired fibrinolysis, collectively contribute to the elevated thrombotic risk observed in TB patients admitted to the intensive care unit. A total of 168 patients with tuberculosis were enrolled in this study. The analysis revealed that advanced age and prolonged APTT were independent predictors of thrombus formation. Although advanced age has been widely recognized as a risk factor for venous thromboembolism in critically ill populations, the independent prothrombotic role of APTT—a parameter traditionally used to assess bleeding risk—merits further investigation (14–16). Under the inflammatory state of pulmonary tuberculosis, the intrinsic coagulation pathway is abnormally and persistently activated, continuously consuming intrinsic coagulation factors, ultimately leading to prolonged APTT, which is consistent with findings reported in the literature (6, 17). No clinically significant bleeding events were documented in our cohort, despite the presence of prolonged APTT in the thrombosis group, suggesting that APTT prolongation in this setting may reflect inflammation-driven coagulopathy rather than a true hemorrhagic tendency. Although univariate analysis demonstrated a significant association between elevated DDR levels and thrombus formation, this independent predictive value was attenuated after multivariable adjustment for age and coagulation parameters, suggesting that the effect of DDR is largely confounded by age and coagulation parameters. It should be noted that due to the retrospective design, the temporal relationship between blood sampling and thrombus detection could not be uniformly established. Therefore, the associations between laboratory parameters and thrombosis should be interpreted as cross-sectional rather than prospectively predictive.

Notably, fungal infection did not reach statistical significance in the univariate analysis; however, after multivariable adjustment, it emerged as a strong independent risk factor for thrombus formation (OR ≈ 3.2). This statistical phenomenon may be attributed to negative confounding effects exerted by age or APTT. Fungal infection can trigger intense inflammatory cascades and cause endothelial damage, thereby promoting thrombus formation in immunocompromised hosts (18, 19). In stark contrast, the present study found that bacterial infection was negatively correlated with thrombotic risk, exhibiting a seemingly paradoxical protective effect, the mechanism of which remains to be elucidated. Possible explanations include: (1) patients with bacterial infection are subject to more extensive biomarker monitoring, enabling clinicians to detect the infection earlier and implement more aggressive anticoagulant prophylaxis; and (2) infection-related inflammation of specific types may exert a modulatory effect on the coagulation–fibrinolysis balance (20). These findings suggest that the association between bacterial infection and thrombus formation is highly complex and warrants further validation in studies with larger sample sizes.

To evaluate the incremental predictive performance of inflammatory markers, we constructed a base logistic regression model incorporating age, APTT, and bacterial and fungal infection status, which yielded an AUC of 0.753, demonstrating moderate discriminatory ability. Subsequently, CRP, DDR, IL-6 were incorporated into the model individually or in combination, resulting in only a marginal increase in the AUC to 0.764. DeLong test results indicated that none of the extended models achieved a statistically significant improvement in AUC compared with the base model (all *p* > 0.05). This lack of significant incremental value may be attributed to insufficient statistical power arising from collinearity between inflammatory markers and the variables in the base model, as well as the inherent heterogeneity of thrombotic mechanisms in patients with tuberculosis. Furthermore, the exclusion of key covariates—such as duration of bed rest, central venous catheterization, and anti-tuberculosis treatment regimens—from the model may have diminished the additional predictive contribution of these markers. Nevertheless, this base model still demonstrates acceptable utility for clinical risk stratification.

From the perspectives of public health strategy and clinical practice, the findings of this study carry important implications for optimizing intensive care guidelines for tuberculosis and allocating medical resources. First, the parsimonious model comprising age, APTT, and co-infection type (bacterial or fungal) provides a practical tool for thrombotic risk stratification in tuberculosis intensive care unit (TB-ICU) patients where biomarker testing is limited. Given that the addition of CRP, DDR, or IL-6 did not significantly improve the discriminatory ability of the model, it is suggested that standardized thrombotic screening in critically ill tuberculosis patients need not rely on complex inflammatory testing panels; clinicians can identify high-risk individuals using routinely available laboratory parameters. Second, the age-dependence of thrombotic risk underscores the urgency of developing age-specific prophylactic strategies. Our stratified analysis revealed that in patients under 70 years of age, age was the sole independent predictor, whereas in the elderly population, multiple age-related factors were so intricately intertwined that it was difficult to isolate a single independent risk factor. Therefore, future clinical guidelines recommending pharmacological thromboprophylaxis should consider establishing age-specific thresholds, moving away from the suboptimal “one-size-fits-all” approach. Third, the multivariable model revealed that fungal and bacterial co-infections play distinctly different roles in thrombus formation, implying the need for infection-type-specific prophylactic recommendations. If prospective cohort studies confirm the high-risk profile of fungal infection, guideline developers may delineate it as a high-risk subgroup, implementing more intensive surveillance and earlier initiation of anticoagulation therapy. Conversely, the protective effect observed with bacterial infection suggests that certain co-infections may attenuate the host’s hypercoagulable state—a hypothesis that warrants further investigation before translation into clinical practice. Finally, this study confirmed that CRP serves as a reliable and independent surrogate marker for IL-6-driven inflammatory responses in this population, offering a cost-effective alternative for assessing inflammatory burden. In settings where IL-6 testing is unavailable, CRP can function as an effective indicator of the inflammatory component of thrombotic risk, thereby guiding decisions on adjunctive anti-inflammatory therapy and enabling the concentration of limited financial resources on interventions that improve patient outcomes. Incorporating these insights into revisions of TB-ICU management guidelines will ultimately enhance the precision of thromboprophylaxis, reduce unnecessary laboratory expenditure, and improve clinical outcomes for patients.

Regarding the determinants of IL-6 levels, only CRP remained independently associated with IL-6 levels after adjustment for age, coagulation parameters, and DDR. This finding is biologically plausible, as it is well established that IL-6 is the key upstream cytokine driving hepatic CRP synthesis. The strong correlation between IL-6 and CRP across various inflammatory conditions has been well documented in the literature (21–23). The present study extends this association to the tuberculosis population, further corroborating that CRP levels serve as a valid indicator of IL-6-driven inflammatory status. However, it must be noted that CRP and IL-6 are not entirely interchangeable, given that IL-6 is also broadly involved in physiological processes such as thrombopoiesis and B-lymphocyte differentiation (24, 25).

## 5 Limitations

Several limitations should be acknowledged. First, the single-center, retrospective design may introduce selection bias and limit generalizability. Second, the moderate sample size (*n* = 168) may have reduced the statistical power to detect small effect sizes or minor improvements in AUC, and the limited number of deaths (10 in the thrombosis group vs. 2 in the non-thrombosis group) precluded formal mortality analysis. Third, residual confounding cannot be excluded, particularly regarding the unexpected protective effect of bacterial infection. Fourth, several clinically relevant variables—including bed rest days, central venous catheterization, anticoagulant therapy, anti-tuberculosis drug regimens, disease duration, specific sites of extrapulmonary tuberculosis, body mass index, and indications for ICU admission—were not systematically documented or could not be reliably extracted from electronic medical records, which precluded formal adjustment for these factors in the multivariate model. Additionally, the temporal relationship between thrombosis onset and anti-tuberculosis medication could not be established. Fifth, the cross-sectional nature of the study precludes causal inference, and the predictive model requires external validation in an independent cohort before clinical implementation. Furthermore, optimal cut-off values for laboratory parameters were not determined, as the study aim was risk factor identification rather than clinical prediction tool development. Future prospective studies with larger sample sizes should systematically collect these variables, incorporate standardized disease severity scoring, and perform dedicated mortality analyses to validate and extend these findings.

## 6 Conclusion

Age and APTT are independent risk factors for thrombus formation in patients with tuberculosis. Concurrent fungal infection may confer additional thrombotic risk, whereas bacterial infection exhibited a paradoxical protective effect, the mechanism of which warrants further investigation. The parsimonious model constructed based on age, APTT, and co-infection status demonstrated moderate predictive performance (AUC = 0.753), and the incorporation of CRP, DDR, or IL-6 did not significantly improve its discriminatory ability. Furthermore, CRP was strongly correlated with IL-6 and may serve as a practical surrogate marker for IL-6-driven inflammatory responses in resource-limited settings. Large-scale prospective studies incorporating a broader range of clinical and laboratory variables are needed to validate and refine these findings.

## Data Availability

The original contributions presented in the study are included in the article/supplementary material, further inquiries can be directed to the corresponding author.

## References

[1] Hurtado J Coitinho C Nin N Buroni M Hurtado FJ Robello C . Clinical and epidemiological features of tuberculosis isolated from critically ill patients. Rev Argent Microbiol. (2022) 54:43–7. doi: 10.1016/j.ram.2021.02.011, 34001412

[2] de Vasconcellos K Ramjathan P Singh D. The utility of point-of-care urinary lipoarabinomannan testing for the diagnosis of tuberculosis in critically ill patients: a prospective observational study. BMC Infect Dis. (2021) 21:281. doi: 10.1186/s12879-021-05979-y, 33740905 PMC7980562

[3] Mitroi DM Balteanu MA Cioboata R Vlasceanu SG Zlatian OM Catana OM . Hypercoagulability in tuberculosis: pathophysiological mechanisms, associated risks, and advances in management-a narrative review. J Clin Med. (2025) 14:762. doi: 10.3390/jcm14030762, 39941433 PMC11818899

[4] Suresh PS Mathivanan KMR. Disseminated tuberculosis complicated by pulmonary thromboembolism. Cureus. (2025) 17:e89662. doi: 10.7759/cureus.89662, 40926907 PMC12415404

[5] Mitroi DM Vlasceanu SG Zlatian OM Olteanu M Catană OM Mititelu RR . Hypercoagulability in pulmonary tuberculosis: reduced protein C and free protein S predict pulmonary embolism-evidence from a prospective Romanian cohort. J Clin Med. (2026) 15:1903. doi: 10.3390/jcm15051903, 41827320 PMC12985418

[6] Danwang C Bigna JJ Awana AP Nzalie RN Robert A. Global epidemiology of venous thromboembolism in people with active tuberculosis: a systematic review and meta-analysis. J Thromb Thrombolysis. (2021) 51:502–12. doi: 10.1007/s11239-020-02211-7, 32627124

[7] Sharif-Kashani B Azimi M Tabarsi P Sadr M Shirzadi S. Investigation of two general venous thromboembolism risk-stratification models in predicting venous thromboembolic events in TB patients. Int J Mycobacteriol. (2022) 11:83–7. doi: 10.4103/ijmy.ijmy_252_21, 35295028

[8] Purayil NK Sirajudeen J Al Arbi KM Baghi MA Zahid M. Venous thromboembolism: an unusual presentation of pulmonary tuberculosis. Cureus. (2021) 13:e14092. doi: 10.7759/cureus.14092, 33907638 PMC8065094

[9] Nan GY Fei H Zhen W Yun DT. Risk factors associated with venous thromboembolism in tuberculosis: a case control study. Clin Respir J. (2022) 16:835–41. doi: 10.1111/crj.13555, 36344481 PMC9716713

[10] Scherger S Henao-Martínez A Franco-Paredes C Shapiro L. Rethinking interleukin-6 blockade for treatment of COVID-19. Med Hypotheses. (2020) 144:110053. doi: 10.1016/j.mehy.2020.110053, 32758889 PMC7320867

[11] Ma X Xiao S Tan A Liu A Xiao J. Association of serum Interleukin-6 with dysregulated lipid metabolism and nutritional status in patients with pulmonary tuberculosis: a case-control study. BMC Infect Dis. (2026) 26:594. doi: 10.1186/s12879-026-12823-8, 41680666 PMC13005510

[12] National Health and Family Planning Commission of the People's Republic of China. WS 288—2017 Diagnosis of Pulmonary tuberculosis. Beijing: China Standards Press (2017).

[13] Barbar S Noventa F Rossetto V Ferrari A Brandolin B Perlati M . A risk assessment model for the identification of hospitalized medical patients at risk for venous thromboembolism: the Padua prediction score. J Thromb Haemost. (2010) 8:2450–7. doi: 10.1111/j.1538-7836.2010.04044.x, 20738765

[14] Huang CB Hong CX Xu TH Zhao DY Wu ZY Chen L . Risk factors for pulmonary embolism in ICU patients: a retrospective cohort study from the MIMIC-III database. Clin Appl Thromb Hemost. (2022) 28:10760296211073925. doi: 10.1177/10760296211073925, 35043708 PMC8796081

[15] Klok FA Kruip MJHA van der Meer NJM Arbous MS Gommers DAMPJ Kant KM . Incidence of thrombotic complications in critically ill ICU patients with COVID-19. Thromb Res. (2020) 191:145–7. doi: 10.1016/j.thromres.2020.04.013, 32291094 PMC7146714

[16] Erben Y Franco-Mesa C Gloviczki P Stone W Quinones-Hinojoas A Meltzer AJ . Deep vein thrombosis and pulmonary embolism among hospitalized coronavirus disease 2019-positive patients predicted for higher mortality and prolonged intensive care unit and hospital stays in a multisite healthcare system. J Vasc Surg Venous Lymphat Disord. (2021) 9:1361–1370.e1. doi: 10.1016/j.jvsv.2021.03.009, 33836287 PMC8023789

[17] Suryakusumah L Tabri NA Saleh S Bakri S Kasim H Benyamin AF . Hemostatic parameters in pulmonary tuberculosis patients after intensive phase treatment. Caspian J Intern Med. (2021) 12:294–8. doi: 10.22088/cjim.12.3.294, 34221279 PMC8223056

[18] Ralaizanaka BM Razafindrazoto CI Bolot E Bors G Housson-Wetzel S Razafimahefa SH . Gastrointestinal Mucormycosis-induced massive lower gastrointestinal bleeding, rectal perforation, and pulmonary embolism: a long diagnostic pathway in a case report. Clin Exp Gastroenterol. (2022) 15:145–51. doi: 10.2147/CEG.S373728, 35983373 PMC9381012

[19] Deshmukh H Speth C Sheppard DC Neurauter M Würzner R Lass-Flörl C . Aspergillus-derived Galactosaminogalactan triggers complement activation on human platelets. Front Immunol. (2020) 11:550827. doi: 10.3389/fimmu.2020.550827, 33123129 PMC7573070

[20] Carestia A Godin LC Jenne CN. Step up to the platelet: role of platelets in inflammation and infection. Thromb Res. (2023) 231:182–94. doi: 10.1016/j.thromres.2022.10.001, 36307228

[21] Cruz-Ávila J Hernández-Pérez E González-González R Bologna-Molina R Molina-Frechero N. Periodontal disease in obese patients; Interleukin-6 and C-reactive protein study: a systematic review. Dent J (Basel). (2022) 10:225. doi: 10.3390/dj10120225, 36547041 PMC9777236

[22] Lee EH Lee KH Song YG Han SH. Discrepancy of C-reactive protein, Procalcitonin and Interleukin-6 at hospitalization: infection in patients with Normal C-reactive protein, Procalcitonin and high Interleukin-6 values. J Clin Med. (2022) 11:7324. doi: 10.3390/jcm11247324, 36555941 PMC9783053

[23] Stanimirovic J Radovanovic J Banjac K Obradovic M Essack M Zafirovic S . Role of C-reactive protein in diabetic inflammation. Mediat Inflamm. (2022) 2022:1–15. doi: 10.1155/2022/3706508, 35620114 PMC9129992

[24] Webb CE Vautrinot J Hers I. IL-6 as a mediator of platelet hyper-responsiveness. Cells. (2025) 14:766. doi: 10.3390/cells14110766, 40497942 PMC12153796

[25] Linge I Tsareva A Kondratieva E Dyatlov A Hidalgo J Zvartsev R . Pleiotropic effect of IL-6 produced by B-lymphocytes during early phases of adaptive immune responses against TB infection. Front Immunol. (2022) 13:750068. doi: 10.3389/fimmu.2022.750068, 35154093 PMC8828505

